# Gasdermin D mediates host cell death but not interleukin-1β secretion in *Mycobacterium tuberculosis*-infected macrophages

**DOI:** 10.1038/s41420-021-00716-5

**Published:** 2021-10-30

**Authors:** Sebastian J. Theobald, Jessica Gräb, Melanie Fritsch, Isabelle Suárez, Hannah S. Eisfeld, Sandra Winter, Maximilian Koch, Christoph Hölscher, Manolis Pasparakis, Hamid Kashkar, Jan Rybniker

**Affiliations:** 1 Department I of Internal Medicine, University of Cologne, 50937 Cologne, Germany; 2 Center for Molecular Medicine Cologne (CMMC), University of Cologne, 50931 Cologne, Germany; 3 Excellence Cluster on Cellular Stress Responses in Aging-Associated Diseases (CECAD), University of Cologne, 50931 Cologne, Germany; 4 Institute for Medical Microbiology, Immunology and Hygiene (IMMIH), University of Cologne, 50935 Cologne, Germany; 5 German Center for Infection Research (DZIF), Partner Site Bonn-Cologne, Cologne, Germany; 6 Division of Infection Immunology, Research Center Borstel, 23845 Borstel, Germany; 7 German Center for Infection Research (DZIF), Partner Site Borstel, 23845 Borstel, Germany; 8 Institute for Genetics, University of Cologne, 50674 Cologne, Germany

**Keywords:** Cell death and immune response, Tuberculosis, Inflammasome

## Abstract

Necrotic cell death represents a major pathogenic mechanism of *Mycobacterium tuberculosis* (*Mtb*) infection. It is increasingly evident that *Mtb* induces several types of regulated necrosis but how these are interconnected and linked to the release of pro-inflammatory cytokines remains unknown. Exploiting a clinical cohort of tuberculosis patients, we show here that the number and size of necrotic lesions correlates with IL-1β plasma levels as a strong indicator of inflammasome activation. Our mechanistic studies reveal that *Mtb* triggers mitochondrial permeability transition (mPT) and subsequently extensive macrophage necrosis, which requires activation of the NLRP3 inflammasome. NLRP3-driven mitochondrial damage is dependent on proteolytic activation of the pore-forming effector protein gasdermin D (GSDMD), which links two distinct cell death machineries. Intriguingly, GSDMD, but not the membranolytic mycobacterial ESX-1 secretion system, is dispensable for IL-1β secretion from *Mtb*-infected macrophages. Thus, our study dissects a novel mechanism of pathogen-induced regulated necrosis by identifying mitochondria as central regulatory hubs capable of delineating cytokine secretion and lytic cell death.

## Introduction

*Mycobacterium tuberculosis* (*Mtb*), the causative agent of tuberculosis (TB) is a leading cause of morbidity and mortality. *Mtb* is highly dependent on the human as a host and has developed different immune modulation and evasion mechanisms to survive [1]. A major mechanism of pathogenesis in *Mtb* is the induction of necrotic host cell death, which leads to bacterial spread, hyperinflammation, and tissue damage [2].

Necrosis has long been described as an unregulated and undruggable process [3]. However, in the past decade various regulated forms of necrosis have been identified, such as necroptosis, ferroptosis, and pyroptosis [4]. Surprisingly, several of these cell death pathways have been independently associated with *Mtb* infection [5–13].

A key virulence factor driving host cell death in mycobacterial infections is the ESX-1 secretion system, a bacterial type VII secretion system [14]. ESX-1 is strictly required for phagosomal escape and induction of necrotic cell death most likely by exploiting membranolytic activities of one or several ESX-1 substrates [15]. Genetic deletion of the ESX-1-coding gene locus leads to substantial attenuation of *Mtb* [16, 17].

How and why *Mtb* and ESX-1 trigger different modes of necrotic death and whether the underlying pathways are interconnected remains unknown. In-depth molecular knowledge would provide opportunities for novel targets in host-directed and anti-virulence treatment approaches, a vivid and rapidly evolving research topic [18]. We have recently identified a mitochondria-mediated necrotic cell death pathway, which involves opening of the mitochondrial permeability transition pore (mPTP) in a hexokinase II (HKII)-dependent manner, resulting in ATP depletion and necrosis upon *Mtb* infection [7]. Permeabilization of the inner mitochondrial membrane induces mitochondrial permeability transition (MPT), which allows the opening of the mPTP and results in disruption of the mitochondrial membrane potential (ΔΨm) via an influx of ions and a loss of mitochondrial integrity [19]. However, the upstream events initiating mPTP opening in response to *Mtb* infection are unknown.

*Mtb* infection has also been shown to induce pyroptotic cell death by utilizing the NLRP3 inflammasome [20]. Inflammasomes are macromolecular complexes formed in response to pathogen-associated molecular patterns (PAMPs) and danger-associated molecular patterns that drive caspase-dependent maturation of the pro-inflammatory cytokines interleukin (IL)−1β and IL-18, and cleave gasdermin D (GSDMD), a pore-forming multimeric protein [21, 22]. GSDMD-induced membrane disruption has been viewed as a central determinant of IL-1β and IL-18 release and pyroptotic cell death [23]. The specific triggers of inflammasome activation and pyroptotic cell death are only fragmentarily described. Potassium efflux has been identified as the common initiating event for canonical and non-canonical NLRP3 pathways [24]. In parallel, there is growing evidence that mitochondrial damage may play a role in NLRP3 inflammasome and gasdermin activation [25, 26]. However, whether mitochondrial damage is a prerequisite for NRLP3 inflammasome formation and pyroptosis or rather a consequence of inflammatory cell death remains unknown.

In this study, we investigated the role of MPT in *Mtb*-mediated host cell necrosis. Our mechanistic studies identify mPTP opening as an early, highly regulated event, which requires activation of the NLRP3 inflammasome. Abrogation of NLRP3-dependent GSDMD cleavage preserves mitochondrial integrity but has no impact on secretion of the pro-inflammatory cytokine IL-1β.

## Results

### *Mtb* infection leads to pronounced mitochondrial damage

**Fig. 1:**
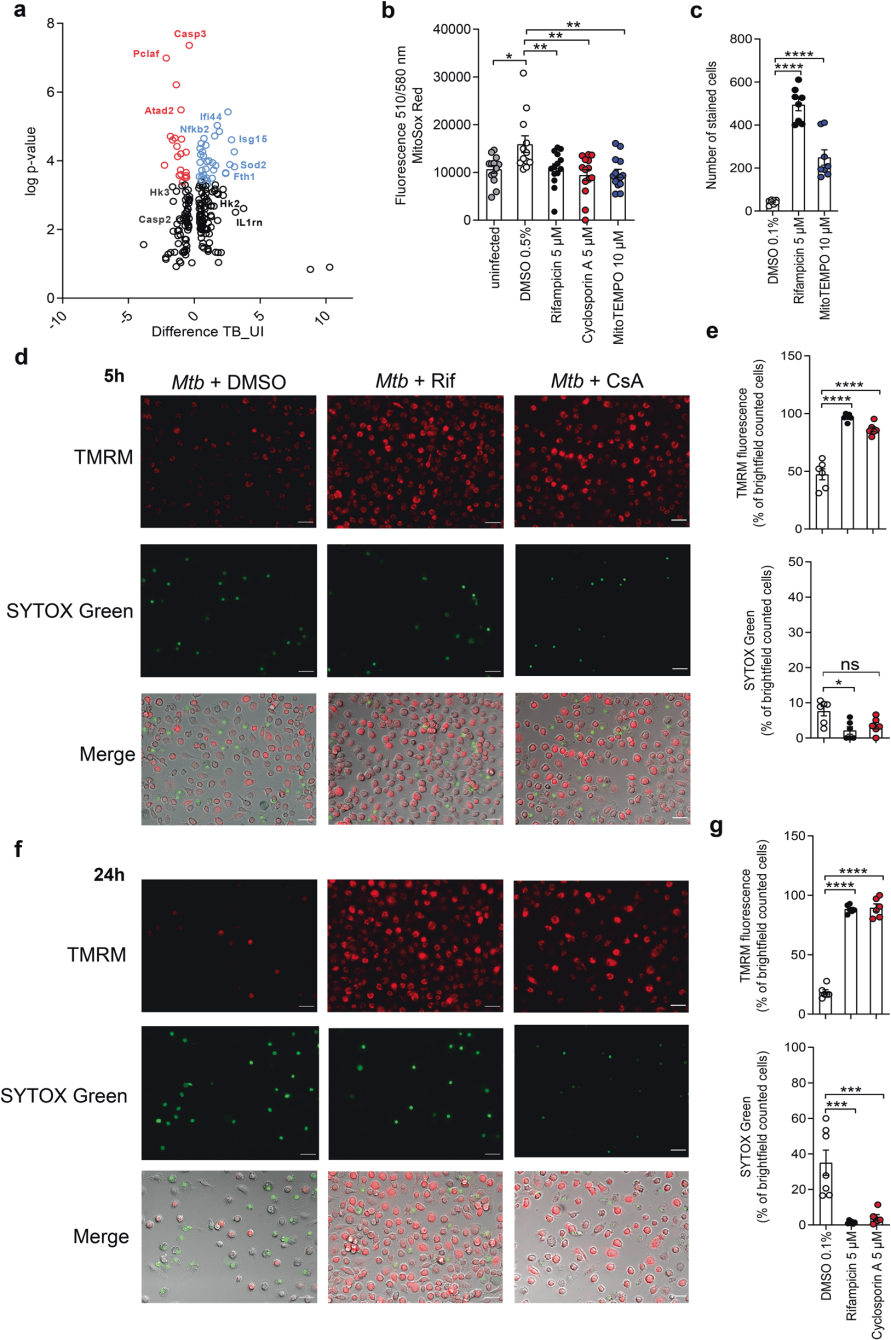
*Mtb* induces mitochondrial damage. **a** Proteome analysis by mass spectrometry of whole-cell lysates of uninfected and *Mtb*-infected J774.2 macrophages showing differentially regulated proteins (macrophage; *n* = 3; black dots = lower log *p* value and difference; red = upregulated genes; blue = downregulated genes). **b** Quantification of mitochondrial ROS in *Mtb*-infected MRC-5 lung fibroblasts (MOI 10) using MitoSOX Red 24 h post infection (*n* = 16). **c** Survival of primary human macrophages infected with *Mtb* (MOI 1) and treated with the superoxide scavenger MitoTEMPO (10 µM). Cells were stained with 4’,6-diamidino-2-phenylindole (DAPI) and the number of surviving cells was determined 48 h post infection (*n* = 8). Data from two experiments with multiple replicates are shown. Results are expressed as mean ± SEM. Analysis was done using one-way ANOVA and Bonferroni post-test (*p ≤ 0.05; **p ≤ 0.001; ****p ≤ 0.0001). **d**–**g** Fluorescence microscopy of primary human macrophages infected with *Mtb* (MOI 1) and treated with cyclosporin A (5 µM). Macrophages were stained with tetramethyl rhodamine (TMRM) and SYTOX Green **d**, **e** 5 h and **f**, **g** 24 h post infection. The number of stained cells was determined in relation to the number of brightfield (BF) counted cells (*n* = 6) (**e**, **g**). Representative images of *Mtb*-infected primary human macrophages stained with TMRM and SYTOX Green **c** 5 h and **e** 24 h post infection (scale bar: 100 μm). Data from two experiments with multiple replicates are shown. Results are expressed as mean ± SEM. Analysis was done using one-way ANOVA with Bonferroni post-test (ns, not significant; *p ≤ 0.05; **p ≤ 0.01; ***p ≤ 0.001; ****p ≤ 0.0001).

*Mtb* induces necrotic cell death by manipulation of the mitochondrial membrane integrity and opening of the mPTP [7]. However, the initiating events leading to mPTP opening in *Mtb* infection are poorly understood. To better define the extent and timing of mitochondrial damage, we first analyzed mitochondrial and cytosolic proteomes of *Mtb*-infected and uninfected macrophages by mass spectrometry. We detected 62 proteins that were altered in response to the infection, which are associated with host cell death and mitochondrial damage (Fig. 1a and Supplementary Fig. 1a). The mitochondrial proteins such as NADH dehydrogenases, succinate dehydrogenases, cytochrome *c*, and superoxide dismutase were upregulated in *Mtb*-infected cells. In addition, host proteins associated with cell death were caspase-1, caspase-3, and PYCARD, which have also been linked to mitochondrial damage. Furthermore, our proteome analysis revealed differential regulation of HKII, which supports our recent findings on the involvement of HKII in mPTP opening upon TB infection (Fig. 1a) [7].

Two major molecular indicators of mitochondrial damage and mPTP opening are the increased production of mitochondrial reactive oxygen species (mROS) and the accumulation of intracellular calcium (Ca^2+^). We show that *Mtb* triggered production and release of mROS from infected cells using MitoSOX Red. Quantification of mROS is challenging and requires suitable controls. For this reason, we show that mROS production could be abrogated by treatment of the infected cells with three different inhibitors: the mPTP and cyclophilin D inhibitor cyclosporin A (CsA), the superoxide scavenger MitoTEMPO, and rifampicin, all of which gave significant results (Fig. 1b). In addition, cell death was significantly reduced in MitoTEMPO-treated, *Mtb*-infected primary human macrophages (Fig. 1c). The intracellular Ca^2+^ homeostasis is primarily regulated by the endoplasmic reticulum and mitochondria [27]. Uptake of Ca^2+^ into mitochondria is a potent trigger for mPTP, which can be prevented by blocking the mitochondrial calcium uniporter (MCU) [28]. We show that chemical inhibition of the MCU using Ru-360 results in increased host cell survival upon *Mtb* infection. Similar effects were seen for the combination of Ru-360 and MitoTEMPO (Supplementary Fig. 1b, c, d). Ca^2+^ flux and mROS release further display considerable mitochondrial dysregulation in response to infection with *Mtb*.

### Mitochondrial damage is an early event preceding disruption of the plasma membrane

Mitochondrial damage can either be the initiating event of cell death or it occurs as a consequence following cell injury. To clarify whether mitochondrial depolarization precedes cell membrane injury and host cell death upon *Mtb* infection, we conducted immunofluorescent staining of primary human macrophages with the ΔΨm indicator tetramethyl rhodamine methyl ester (TMRM) and SYTOX Green dead cell stain, which penetrates compromised cell membranes. As early as 5 h post infection, we detected a significant loss of TMRM positivity upon *Mtb* infection of primary human macrophages compared to CsA-treated *Mtb*-infected macrophages, while only a few cells stained positive for SYTOX Green at this time point (Fig. 1d, e). Rifampicin, a potent bactericidal drug that rapidly inhibits intracellular growth and survival of *Mtb*, was used as a control. At a later time point of infection (24 h), we saw a nearly complete loss of TMRM positivity and an increase in SYTOX Green-stained cells (Fig. 1f, g). However, 65% of cells remained SYTOX negative (Fig. 1g). These data show that mitochondrial damage precedes disruption of cell membrane integrity and host cell death in *Mtb*-infected macrophages.

### Overexpression of Bcl-2 impacts host cell survival in infected macrophages

**Fig. 2:**
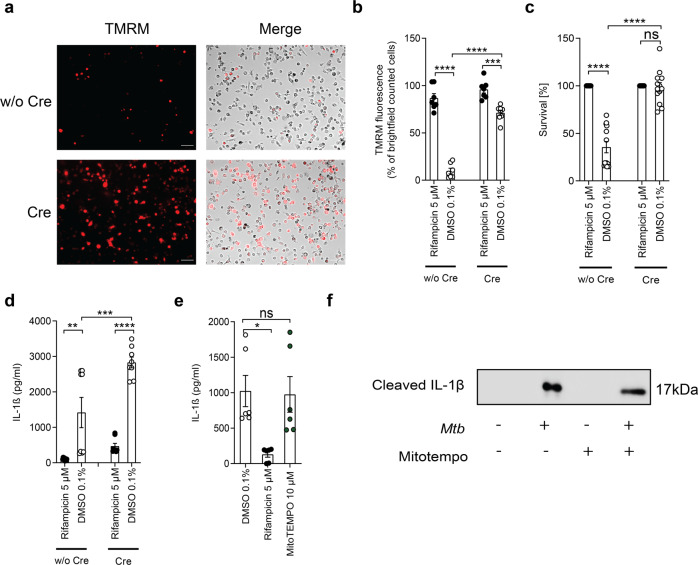
Manipulation of regulators of the mPTP reduces mitochondrial damage. **a** Representative images and **b** quantification of *Mtb*-infected Bcl-2-overexpressing BMDMs treated with or without (w/o) Cre and stained with TMRM 24 h post infection (scale bar: 100 μm; *n* = 7). **c** Survival of bone-marrow derived macrophages (BMDMs) from Bcl-2-overexpressing mice treated with or w/o Cre to induce Bcl-2 overexpression ex vivo. Viability of *Mtb*-infected BMDM (MOI 3) was quantified 24 h post infection using DAPI staining. Rifampicin-treated BMDMs were used as controls and their survival was set to 100% (*n* = 13). Data from two experiments with multiple replicates are shown. **d** Quantification of IL-1β concentration in the supernatant of Bcl-2-overexpressing BMDMs (*n* = 6). **e** Quantification of IL-1β concentration in the supernatant of *Mtb*-infected THP-1 cells treated with or without MitoTEMPO (10 µM; *n* = 4). **f** Detection of cleaved IL-1β in the supernatant of *Mtb*-infected THP-1 treated with or without MitoTEMPO by western blot analysis (representative example of two experiments); same amount of cells were seeded for each condition, and the same amount of supernatant was precipitated and loaded. Results are expressed as mean ± SEM. Analysis was done using one-way ANOVA with Bonferroni post-test (*p ≤ 0.05; **p ≤ 0.01; ***p ≤ 0.001; ****p ≤ 0.0001).

In the past decade, a regulatory network has been linked to tight mPTP control. The anti-apoptotic Bcl-2 protein is known to maintain ΔΨm by directly interacting with components of the mPTP representing an important gatekeeper of pore opening [29]. In order to explore the impact of mitochondrial integrity on *Mtb* infection, we isolated bone marrow-derived macrophages (BMDMs) from mice conditionally overexpressing Bcl-2 after Cre-mediated gene recombination [30, 31]. We show that Bcl-2 efficiently prevents disruption of the ΔΨm, which is indicated by the preservation of TMRM in infected cells (Fig. 2a, b). Furthermore, Bcl-2 overexpression significantly increased host cell survival in *Mtb*-infected BMDMs (Fig. 2c and Supplementary Fig. 1e). Collectively, these data indicate that the inhibition of mitochondrial membrane damage and mPTP opening by manipulation of key regulators significantly increases host cell survival. Our findings clearly link mitochondrial membrane integrity to improved host cell survival, which is abrogated upon *Mtb* infection.

### Inhibition of mPTP-driven cell death fails to block IL-1β secretion

Mitochondrial damage and subsequent release of mitochondrial contents or mROS have been linked to NLRP3 inflammasome activation in other cellular models [25]. To analyze whether the prevention of mitochondrial damage affects inflammasome activation in response to *Mtb* infection, we measured IL-1β secretion in Bcl-2-overexpressing BMDMs. Intriguingly, overexpression of Bcl-2 in infected cells did not block IL-1β secretion despite being highly effective in protecting cells from *Mtb*-induced cell death (Fig. 2d). In addition, blocking mROS with MitoTEMPO results in survival of *Mtb*-infected macrophages (Fig. 1c) but not in inhibition of IL-1β cleavage or secretion (Fig. 2e, f and Supplementary Fig. 1f) suggesting that, in *Mtb*-infected cells, inflammasome activation occurs upstream and independently from mitochondrial damage.

### IL-1β, IL-18, and NLRP3 levels correlate with disease severity and the number of necrotic lesions in vivo

**Fig. 3:**
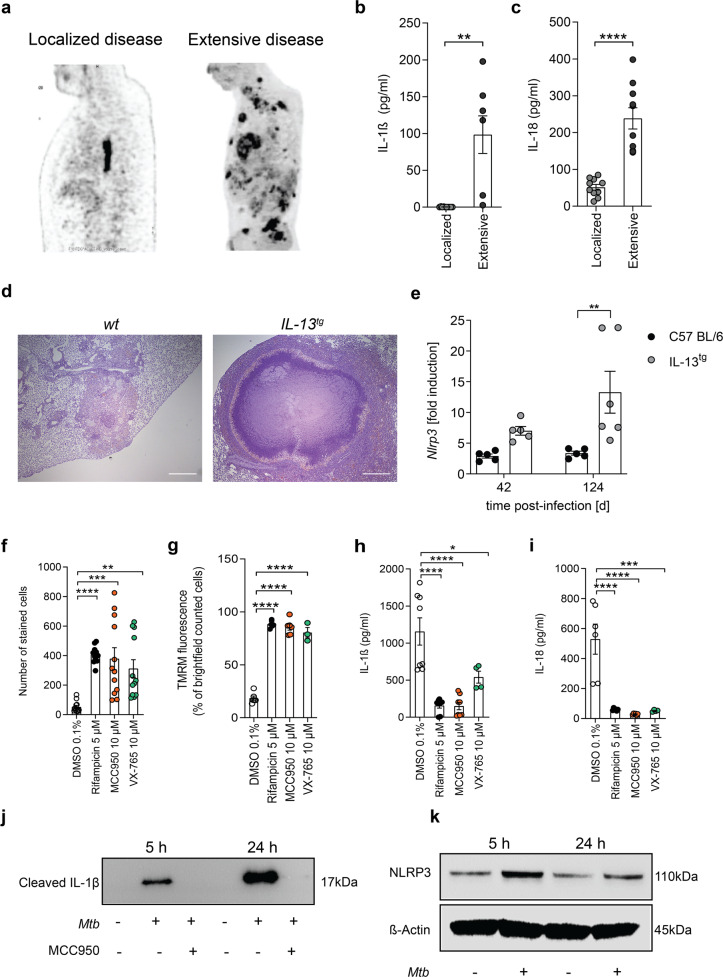
*Mtb*-induced inflammasome activation occurs upstream and independently from mitochondrial damage. **a** ^18^F-FDG PET demonstrating multiple FDG-avid tuberculous lesions in one patient (right side) and with a single PET-positive mediastinal lymph node in a patient on the left side. Lateral chest view. **b** Quantification of IL-1β concentration (pg/ml) in the plasma for TB patients with localized (*n* = 10) and extensive (*n* = 10) tuberculous lesions. **c** Quantification of IL-18 concentration (pg/ml) in the plasma for TB patients with localized (*n* = 10) and extensive (*n* = 10) tuberculous lesions. **d** Representative pictures of H&E-stained lungs explanted from wild-type (*wt*) and *IL-13*^*tg*^ mice infected with *Mtb* (scale bar: 500 µM). **e** RT-q-PCR for detection for NLPR3 expression levels (fold induction) in *Mtb*-infected lungs from *wt* (black circle) and *IL-13*^*tg*^ (gray circle) mice 42 and 124 days post infection. Each dot represents one mouse lung. **f** Survival of *Mtb*-infected primary human macrophages (MOI 1) treated with MCC950 or VX-765 48 h post infection (*n* = 12). **g** Quantification of TMRM stain of *Mtb*-infected primary human macrophages treated with MCC950 or VX-765 (*n* = 3). The number of stained cells was determined in relation to the number of brightfield (BF) counted cells (*n* = 6). Quantification of **h** IL-1β (*n* = 6) and **i** IL-18 (*n* = 8) concentration in the supernatant of *Mtb*-infected primary human macrophages. **j** Detection of cleaved IL-1β in the supernatant of *Mtb*-infected THP-1 by western blot analysis (*n* = 2). **k** Detection of NLRP3 in whole-cell lysates of uninfected and *Mtb*-infected primary human macrophages (*n* = 2). Results are expressed as mean ± SEM. Analysis was done using one-way ANOVA with Bonferroni post-test (ns, not significant; *p ≤ 0.05; **p ≤ 0.01; ***p ≤ 0.001; ****p ≤ 0.0001).

Intrigued by our finding of a sharp delineation of pathogen-induced mitochondrial cell death and cytokine release in our ex vivo infection model, we assessed whether IL-1β and IL-18 levels correlate with severity of disease in TB patients. Extensive forms of the disease are associated with multiple necrotic lesions. We measured IL-1β and IL-18 levels in well-defined cohorts of patients with disseminated disease and patients with localized lymph node TB (Fig. 3a) [32, 33]. Significantly increased levels of IL-1β and IL-18 were detected in the plasma from TB patients with extensive disease when compared to patients presenting with limited disease (Fig. 3b, c).

In order to correlate the extent of *Mtb*-induced necrosis to inflammasome activation in vivo, we exploited transgenic IL-13-overexpressing mice [34], which show human-like lesions and necrotizing granuloma in the lungs upon *Mtb* infection (Fig. 3d). This stands in contrast to *Mtb*-infected wild-type mouse models, which lack granuloma with central necrotic lesions (Fig. 3d) [34]. Reverse transcription quantitative polymerase chain reaction (RT-qPCR) analysis of mouse lung tissue revealed upregulation of NLPR3 upon infection with *Mtb*. In the lungs of *Mtb*-infected IL-13-overexpressing mice, NLRP3 mRNA was highly abundant and expressed to significantly higher levels than in the lungs of wild-type mice at 42 and 124 days post infection (Fig. 3e).

### Inflammasome components are required for *Mtb*-induced host cell death and IL-1β secretion

Having shown that abundance of inflammasome components and associated cytokines correlate with disease severity, we sought to confirm that key components for canonical inflammasome activation mediate IL-1β cleavage and cell death in *Mtb*-infected macrophages in correlation to an early mitochondrial damage.

We first tested whether chemical inhibition of specific components of the NLRP3 inflammasome influences host cell survival following *Mtb* infection. Therefore, we treated primary human macrophages with the NLRP3 inhibitor MCC950 [35] and the caspase-1 inhibitor VX-765 [36]. Both inhibitors potently protected macrophages from *Mtb*-induced cell death (Fig. 3f and Supplementary Fig. 2a) and, more strikingly, diminished *Mtb*-induced mitochondrial damage (Fig. 3g and Supplementary Fig. 2b). We also detected significantly increased levels of IL-1β (Fig. 3h) and IL-18 (Fig. 3i) in the supernatants of *Mtb*-infected primary human macrophages. In line with these results, we detected cleaved IL-1β, a surrogate for activated caspase-1, in the supernatant of *Mtb*-infected THP-1 macrophages, which could be abrogated upon MCC950 treatment (Fig. 3j and Supplementary Fig. 2c). Additionally, we identified increased expression of NLRP3 in *Mtb*-infected primary human macrophages (Fig. 3k and Supplementary Fig. 2d). Hence, these data show that *Mtb* promotes the activation of the NLRP3 inflammasome with subsequent IL-1β/IL-18 secretion in human macrophages.

**Fig. 4:**
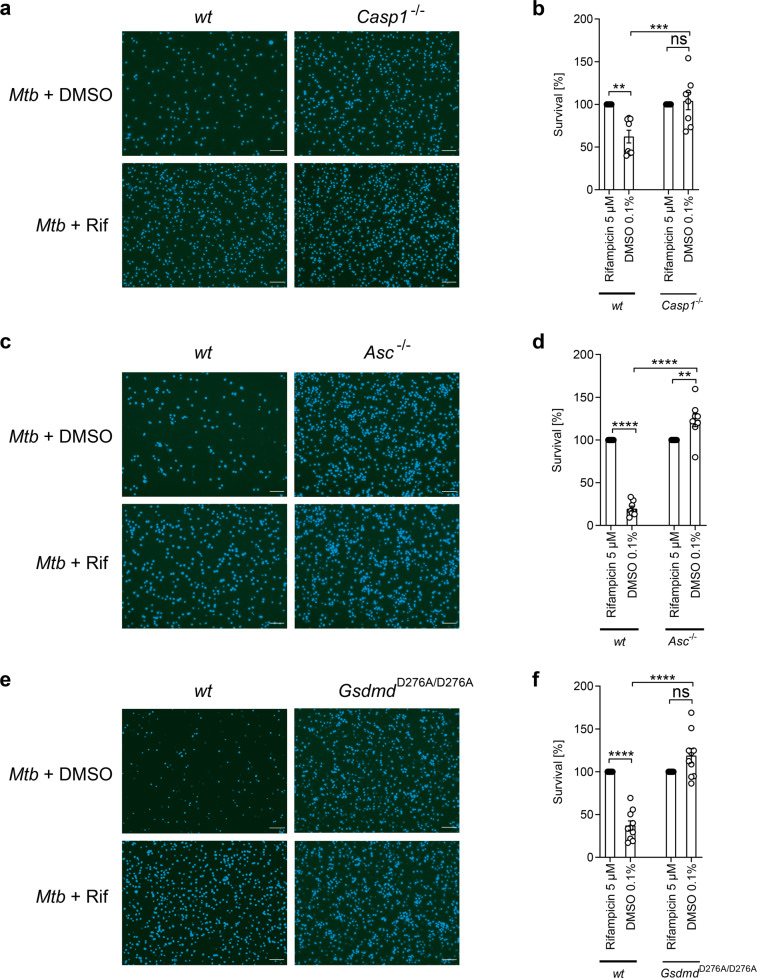
*Mtb* infection induces caspase-1 activation. Survival of BMDM isolated from wild-type (*wt*) and **a**, **b** caspase-1 knockout (*Casp1*^*−/−*^) mice (*n* = 8), **c**, **d** apoptosis-associated speck-like protein containing a CARD knockout (*Asc*^*−/−*^) mice (*n* = 8) or **e**, **f** gasdermin D (*Gsdmd*^*D276A*^) mice (*n* = 10) infected with *Mtb* (MOI 3) was determined 48 h post infection using DAPI staining (scale bar: 100 μm). Data from two experiments with multiple replicates are shown in **a**–**f**. Results are expressed as mean ± SEM. Analysis was done using one-way ANOVA with Bonferroni post-test (ns, not significant; **p ≤ 0.01; ***p ≤ 0.001; ****p ≤ 0.0001).

We further dissected NLRP3 inflammasome-mediated host cell death by analyzing the survival of BMDMs isolated from genetically modified mice. We show that BMDMs isolated from *Caspase-1*^*−/−*^ mice (Fig. 4a, b) and *Asc*^*−/−*^ mice (Fig. 4c, d) were resistant toward *Mtb*-induced host cell death. We also infected BMDMs derived from mice expressing a GSDMD cleavage mutant in which activation by caspase-1 and pore formation is abrogated (*Gsdmd*^*D276A/D276A*^). Cells derived from *Gsdmd*^*D276A/D276A*^ mice were significantly less susceptible to *Mtb*-mediated cell death compared to wild-type cells (Fig. 4e, f). Survival rates of infected *Gsdmd*^*D276A/D276A*^ macrophages were similar to wild-type cells treated with rifampicin. Together, these data underline that *Mtb* promotes a pyroptosis-like cell death involving inflammasome components.

### GSDMD drives mitochondrial damage but not IL-1β secretion in *Mtb*-infected macrophages

Hitherto we had shown that *Mtb* induces cell death by involving early mitochondrial depolarization and, in addition, activation of the NLRP3 inflammasome. We next aimed to connect both *Mtb*-triggered cellular events.

Assuming that activation of the NLRP3 inflammasome is an upstream event, which also triggers mitochondrial damage, treatment with the NLRP3 inhibitor MCC950 should prevent a change in the ΔΨm upon infection. Analysis of TMRM in *Mtb*-infected and MCC950-treated cells indeed revealed a preservation of TMRM staining by MCC950 (Fig. 3g and Supplementary Fig. 2b), indicating that inflammasome inhibition is protective for mitochondria.

**Fig. 5:**
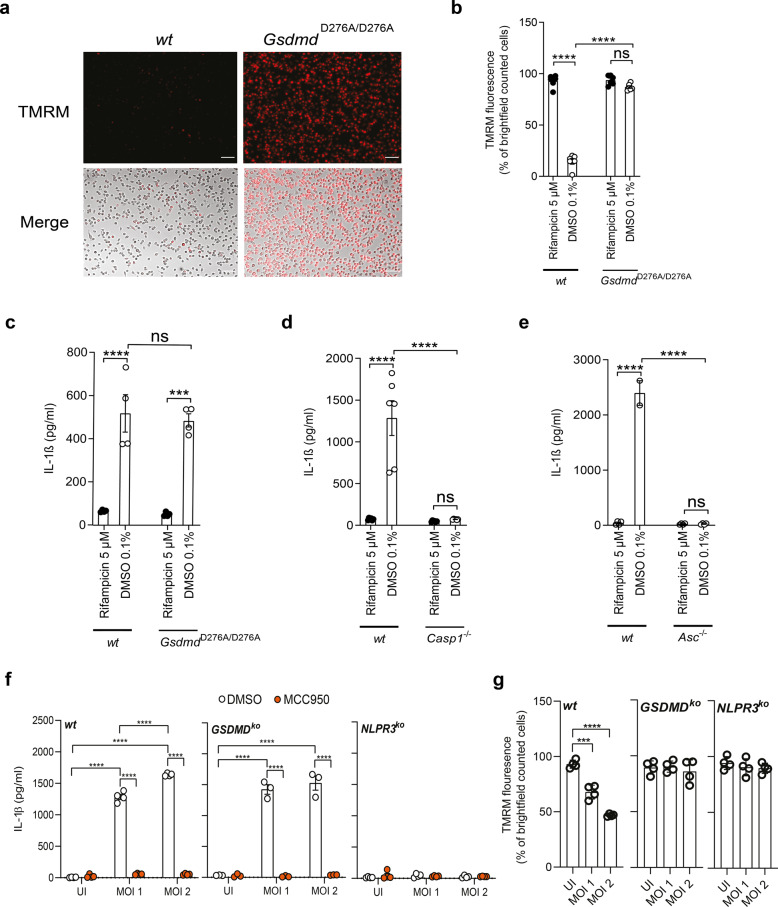
GSDMD is important for mitochondrial integrity but not IL-1β secretion. **a** Representative images and **b** quantification of TMRM in *Mtb*-infected *Gsdmd*^*D276A*^ BMDM 24 h post infection (scale bar: 100 μm; *n* = 6). Data from at least two experiments with multiple replicates are shown. Results are expressed as mean ± SEM. Analysis was done using one-way ANOVA with Bonferroni post-test (ns, not significant; ***p ≤ 0.001; ****p ≤ 0.0001). Quantification of IL-1β concentration (pg/ml) in the supernatant of **c** *Gsdmd*^*D276A*^ (*n* = 4), **d** *casp1*^*−/−*^ (*n* = 6), and **e** *Asc*^−/−^ (*n* = 4) BMDMs 24 h post infection. **f** IL-1β concentration (pg/ml) from THP-1 cells (*n* = 4 for *wt*, *GSDMD*^*ko*^, and *NLPR3*^*ko*^ THP-1 cells), which were infected with *Mtb* for 24 h (MOI as indicated). Before infection, cells were treated for 2 h with DMSO (0.1%, white circle) or MCC950 (10 µM, orange circle). **g** TMRM quantification from THP-1 cells (*n* = 4 for *wt*, *n* = 3 for *GSDMD*^*ko*^, and *n* = 4 for *NLPR3*^*ko*^ THP-1 cells), which were infected with *Mtb* for 24 h (MOI as indicated).

In order to link inflammasome activation and mitochondrial damage, we next investigated the ΔΨm in BMDMs from *Gsdmd*^*D276A/D276A*^ mice using TMRM staining. Intriguingly, in these cells, the ΔΨm was strongly preserved, once more linking inflammasome activation and GSDMD to mPTP opening (Fig. 5a, b). The best-described role of GSDMD is execution of pyroptotic cell death via pore formation in the cellular membrane induced by PAMPs such as lipopolysaccharide (LPS), which subsequently leads to IL-1β secretion. Thus, we quantified IL-1β in the supernatants of LPS-treated or *Mtb*-infected macrophages. As expected, *Gsdmd*^*D276A/D276A*^ BMDMs failed to secrete IL-1β upon treatment with LPS and Nigericin as the inflammasome-activating molecules (Supplementary Fig. 3a). Intriguingly and in contrast to this finding, *Gsdmd*^*D276A/D276A*^ BMDMs strongly secreted IL-1β upon *Mtb* infection where levels were comparable to wild-type BMDMs (Fig. 5c). *Caspase-1*^*−/−*^ (Fig. 5d) and *Asc*^*−/−*^ BMDMs (Fig. 5e) in contrast showed significantly reduced IL-1β levels upon infection, indicating that NLRP3 inflammasome components (ASC) and targets (caspase-1) function upstream of GSDMD and have a potential dual role in cell death and release of pro-inflammatory cytokines. In contrast, GSDMD cleavage seems to play a role in *Mtb*-induced mitochondrial damage, which is independent of inflammasome-mediated IL-1β secretion.

In order to corroborate these findings in human cell systems and to validate knock-out BMDM results, we employed CRISPR–Cas9 technology to generate THP-1 monocyte lines lacking GSDMD and NLPR3 (Supplementary Fig. 3b). Upon infection with *Mtb*, wild-type cells secreted large amounts of IL-1β in a dose-dependent manner (Fig. 5f). Intriguingly, THP-1 *GSDMD* knock-out cells were not defective in IL-1β secretion upon *Mtb* infection, confirming our results of the *Gsdmd*^*D276A/D276A*^ BMDMs (Fig. 5a). In contrast, IL-1β secretion was fully abrogated in *NLPR3* knock-out THP-1 cells. Chemical inhibition with MCC950 abrogated *Mtb*-mediated IL-1β secretion in THP-1 wild-type and *GSDMD* knock-out cell lines (Fig. 5f). LPS and Nigericin, as classical triggers, induced IL-1β secretion in wild-type THP-1 cells, but not in knock-out cells (Supplementary Fig. 2d). We also confirm that both GSDMD and NLRP3 are required for *Mtb*-induced mitochondrial membrane disruption using TMRM staining (Fig. 5g and Supplementary Fig. 2c).

**Fig. 6:**
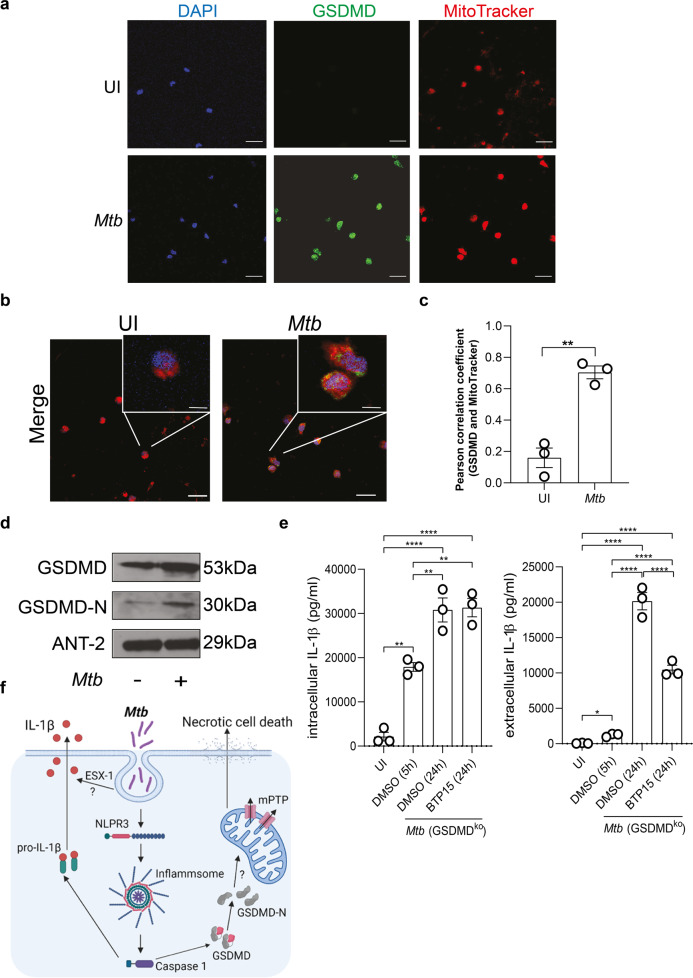
GSDMD is associated with the mitochondria and *Mtb* ESX-1 influences IL-1β secretion in infected macrophages. **a**, **b** Immunofluorescence staining of GSDMD in uninfected (UI) and *Mtb*-infected primary human macrophages. **a** Cells were stained with DAPI (blue), GSDMD (green), and MitoTracker (red) as indicated. **b** Merge pictures of **a**. Images are representative of three individual experiments (scale bar: 50 µM). **c** Quantification of the Pearson correlation coefficient to determine overlapping signals from the GSDMD and MitoTracker staining from **a** and **b**. Standard deviation of the mean is indicated and *T* test with Welsh’s correction was used to determine statistical significance. **≤0.01. **d** Detection of GSDMD in mitochondrial lysates of uninfected and *Mtb*-infected primary human macrophages by western blot analysis. The blot is representative of four individual experiments. **e** IL-1β concentration (pg/ml) from THP-1 GSDMD knock-out cells infected with Mtb (MOI 1) and treated 5 h post infection with BTP-15 (10 µM), as indicated. IL-1β was measured from total cell lysates and the supernatant at 5 h post infection and 24 h post BTP15 treatment. Results are expressed as mean ± SEM. Analysis was done using one-way ANOVA with Bonferroni post-test (ns, not significant; *≤0.05, **≤0.01, ****p ≤ 0.0001). **f** Infection of macrophages by *Mtb* leads to an NLPR3 inflammasome formation and activation with consequent secretion of cleaved IL-1β. Further, *Mtb* infection induces an accumulation of GSDMD-N at the mitochondria, which is associated with mitochondrial damage MPTP opening. BCL-2, as a mitochondrial protein, interferes with mitochondrial damage. Consequently, *Mtb* induces necrotic cell death in macrophages.

Gasdermins have been shown to interact with both the cell membrane and the mitochondrial membrane [37]. We performed confocal microscopic studies using mitochondrial staining with MitoTracker red and staining of GSDMD in infected primary human macrophage (Fig. 6a, b). Pearson correlation analyses revealed a significant colocalization of GSDMD and the mitochondria in *Mtb*-infected macrophages (Fig. 6c). Further, we were able to detect full-length GSDMD and cleaved GSDMD (GSDMD-N) in total protein lysates of mitochondria isolated from *Mtb*-infected primary macrophages (Fig. 6d).

### The mycobacterial ESX-1 secretion system mediates IL-1β secretion

Efficient IL-1β secretion in the absence of GSDMD requires an alternative mechanism for membrane trafficking of this cytokine that lacks a signal sequence. We asked whether the mycobacterial ESX-1 secretion system mediates release of IL-1β in macrophages lacking GSDMD. This bacterial type VII secretion system has been shown to disrupt cell membranes in a contact-dependent manner, which may enable secretion of signaling molecules from infected cells. The recent development of selective ESX-1 inhibitors such as BTP15 [38] allowed us to conditionally shut down secretion of ESX-1 effector proteins 5 h after infection of *GSDMD* knock-out macrophages. At this time point, the NLRP3 inflammasome has already been triggered by *Mtb* and IL-1β levels are elevated in the cytosol (Figs. 3k and 6e). Quantification of IL-1β at 24 h after BTP-15 treatment revealed that chemical inhibition of ESX-1 has significant impact on IL-1β secretion while intracellular IL-1β levels were unaffected and increased to the same levels as observed in untreated cells (Fig. 6e). These data link the ESX-1 secretion system to membrane trafficking of cytokines, which represents a novel function for this important mycobacterial virulence factor.

## Discussion

Cell death is a fundamental feature of life and functions in a multitude of biological processes, such as development, homeostasis, and pathogenesis. In the past decade, a growing number of highly regulated forms of necrosis have been associated with host cell death induced by pathogenic microbes. For *Mtb*, a pathogen with considerable impact on human health, several modes of regulated host cell death have been identified independently and a connecting link has been missing so far [7, 8, 12, 21, 39–42].

By exploiting both human and mouse primary monocytes, we confirm that mitochondria play a crucial role in mediating macrophage necrosis upon mycobacterial infection. Several lines of evidence show profound mitochondrial damage occurring only few hours after phagocytosis of *Mtb*. By dissecting parallel events activating cell death machineries related to the inflammasome and classical pyroptosis, we show that NLRP3-driven macrophage necrosis and mPTP opening are highly linked and mutually dependent.

The interplay between mitochondrial damage, NLPR3 inflammasome activation, and IL-1β secretion has also been the focus of other studies. For instance, two studies by Vince et al. and Chauhan et al. have shown that the mitochondria-related proteins BAK and BAX are able to induce mitochondrial outer membrane permeabilization with subsequent caspase-8-dependent IL-1β secretion, which seems to be independent of NLPR3 and gasdermins. Additionally, Stutz et al. showed that BAK- and BAX-mediated apoptosis plays an essential role in *Mtb*-specific immunity and clearance, whereas non-apoptotic functions of caspase-8 are dispensable for anti-Mtb immunity [43, 44], [13]. In addition, release of mROS and mtDNA by damaged mitochondria has been suggested to induce NLPR3 activation and subsequently IL-1β secretion in several studies. These data, mostly exploiting non-infectious disease models to induce cell stress, clearly link perturbation of mitochondria to cytokine secretion or NLRP3 inflammasome activation [45, 46]. However, the mechanism we dissected for *Mtb*-induced cell death and IL-1β release is fundamentally different. Our data rather indicate that mitochondrial membrane permeabilization is not a prerequisite for NLRP3-dependent pyroptotic cell death but rather a consequence of NLRP3/ASC/caspase-1-associated inflammasome activation (Fig. 6f). Key to this finding was the quantification of IL-1β in the supernatants of genetically modified or chemical inhibitor-treated primary macrophages upon infection with *Mtb*. IL-1β is critical to mediation of inflammation and the host response to infection. We found that classical components of the NLRP3 inflammasome are essential for both *Mtb*-triggered IL-1β secretion and host cell death. However, cell death and IL-1β processing can be uncoupled by preserving mitochondrial integrity in *Mtb*-infected macrophages, whereas NLRP3 activation inevitably results in a pyroptosis-like setting with necrotic cells releasing IL-1β. This clearly indicates that inflammasome formation is not initiated by the mPTP or damaged mitochondria. In fact, our data rather show that, in *Mtb*-infected cells, initiation of the NLRP3 inflammasome complex is required for induction of mitochondrial damage. In line with this assumption, we were able to show that mitochondrial membrane integrity is strongly preserved when inflammasome formation is abrogated.

The current model for both canonical and non-canonical inflammasome activation causing macrophage death involves GSDMD-mediated release of IL-1β through pore formation in the plasma membrane [47]. Our data challenge this model for *Mtb* infection in which GSDMD cleavage/activation is not required for IL-1β release in both human and mouse macrophages. Intriguingly, the phenotype of infected macrophage with mutated GSDMD correlates with the phenotype we found in Bcl-2-overexpressing macrophages: strong abrogation of host cell death but little or no effect on IL-1β processing and secretion. Bcl-2 clearly is a mitochondria-associated protein, which functions as the guardian of mitochondrial membrane integrity. By exploiting a *Gsdmd*^*D276A/D276A*^ cleavage-deficient mutant, we confirm that GSDMD enzymatic processing is required for mitochondrial depolarization and cell death in *Mtb*-infected macrophages. In the infected GSDMD cleavage mutant, the ΔΨm remained almost entirely preserved. Thus, there is a high degree of crosstalk between mitochondrial membrane integrity and GSDMD-driven cell death. Further analyses are required to completely dissect the exact role of GSDMD with regard to disruption of the ΔΨm and macrophage death upon *Mtb* infection. A recent in vivo study revealed that *Mtb*-infected mice lacking GSDMD show histopathological features and lung colony-forming units similar to infected wild-type mice suggesting a minor role of the NLRP3 inflammasome and GSDMD in *Mtb* pathogenesis [13]. While this correlates with our finding of IL-1β secretion occurring independently of GSDMD in infected macrophages, it becomes evident that alternative animal models presenting with human-like necrotic granuloma are required to investigate the exact role of cell death-associated pathways in experimental TB. We show here that inflammasome components such as NLRP3 are regulated to a much higher levels in IL-13 transgenic mice, which better reflect the pathology found in human TB patients. This interesting finding requires further evaluation in the future.

By exploiting the specific phenotype of GSDMD mutant macrophages, we also identified a novel function for the mycobacterial ESX-1 secretion system, which mediates IL-1β release across the plasma membrane following *Mtb* infection. ESX-1 secretes several ill-defined protein substrates, which are required for phagosome permeabilization and translocation of *Mtb* into the cytosol [15]. Membranolytic activity of this system is non-selective and also affects the plasma membrane [48]. To experimentally address whether membrane-disrupting activity plays a role in cytokine release, conditional shutdown of ESX-1 is required since ESX-1 is also essential for NLRP3 inflammasome activation and subsequent processing of caspase-1 substrates. Interestingly, blocking ESX-1 with the anti-virulence compound BTP15 several hours after infection strongly reduced IL-1β secretion without affecting intracellular IL-1β levels. Thus membranolytic activity of ESX-1 is not sufficient to drive necrosis in macrophages with impaired cell death machineries; however, the system clearly has a dual role in both inflammasome activation and membrane trafficking of cytokines, which describes a new feature of this prominent virulence factor. Ou et al. recently described another *Mtb*-derived protein, namely EST12, which causes pyroptosis by binding to RACK1 in macrophages. In line with our findings, these results show that *Mtb* induces host cell pyroptosis in macrophages [49].

In summary, our study describes a novel mechanism of pathogen-induced necrosis and cytokine release by linking two distinct cell death-associated machineries. This mechanism is unique and illustrates how microbial pathogens and associated PAMPs activate similar receptors and pathways leading to diverse outcomes. Exploiting LPS and other virulence determinants, several Gram-negative pathogens activate NOD-like receptor signaling and the NLRP3 inflammasome, which results in classical pyroptosis. We show that the important human pathogen *Mtb*, which presents with a distinct clinical pattern of disease, is capable of exploiting alternative branches of the same pathway. Careful dissection of these unique mechanisms can provide new targets for tailored therapeutic interventions within host-directed and anti-virulence treatment approaches helping to overcome the antibiotic resistance crisis.

## Methods

### Ethics statement

Blood samples were obtained from patients with active TB and from healthy volunteers. The study was approved by the University of Cologne Ethics Committee (18-079). Patients as well as healthy volunteers participated after giving written informed consent.

All animal experiments were performed in accordance with institutional, state, and federal guidelines (approved by the Landesamt fuer Natur, Umwelt und Verbraucherschutz (LANUV) North Rhine-Westphalia, Germany) and the Schleswig-Holstein Ministry of Energy, Agriculture, the Environment, Nature and Digitalization, Germany). *Gsdmd*^*D276A*^ mice were generated by electroporation of C57BL/6N zygotes with 4 μM SpCas9 protein (IDT, #1074181), 4 μM gRNA (5′-CCAGATGGGATTGATGAGGA-3′, IDT) and 10 μM template DNA (5′-TCTGCTCCCTGGATCTACCCGCTCCTAACTCCAGTTCCCCAAGACCTCACAGGCTGCCTGCCCTCTGTCCCTCCAGCTGGCATCGATGAGGAGGAATTAATTGAGGCGGCAGACTTCCAGGGCCTGTTGCTGAGGTGAAGGCTTGCTCC-3, IDT). Ear cuts were genotyped by TaqI restriction digest of the PCR product (5′-ACAAAACAGCTCTTCCCCTT-3′ and 5′-ATTTTACAGGACCAGCCCA-3′). Founders carrying the intended D276A mutation were backcrossed once to a C57BL/6N genetic background. All mice studies were performed after approval by local government authorities (LANUV, NRW, Germany) in accordance with the German animal protection law. Animals were housed in the animal care facility of the University of Cologne under standard pathogen-free conditions with a 12-h light/dark schedule and provided with food and water ad libitum [50]. ASC and caspase-1 knockout mice were recently described [50].

### Small molecules used in this study

Rifampicin was purchased from AppliChem (Darmstadt, Germany). CHIR99021, dimethyl sulfoxide, MCC950, MitoTEMPO, Pifithrin-α, and VX-765 were purchased from Merck (Darmstadt, Germany). CsA was obtained from Cayman Chemicals (Ann Arbor, USA). BTP15 was synthetized as described recently [38].

### Isolation of human macrophages

Peripheral blood mononuclear cells were isolated from human blood after ficoll gradient separation (GE Healthcare) and CD14^+^ cells were isolated using CD14 MicroBeads (Miltenyi Biotec, Bergisch Gladbach, Germany). Cell numbers were determined using a Neubauer chamber by Trypan blue staining, and cells were seeded at a density of 1 × 10^5^ cells per well in 96-well plates (Corning). Monocytes were differentiated into macrophage for 4 days in Roswell Park Memorial Institute (RPMI) 1640 medium (Thermo Fisher Scientific, Waltham, USA) supplemented with 10% heat-inactivated fetal bovine serum (FBS; Thermo Fisher Scientific) and 50 ng/ml human macrophage colony-stimulating factor (M-CSF; Miltenyi Biotec). The growth medium was changed to RPMI supplemented with 10% FBS prior to infection. Macrophages were grown at 37 °C with 5% CO_2_.

### Isolation of mouse macrophages

BMDMs were isolated from mice. Mice were sacrificed by cervical dislocation, and the legs were removed and dissected from adherent tissue. Femurs and tibia were cut off at both ends and the bone marrow was expelled using a 27 g needle. After centrifugation (10 min, 400 × *g*) cells were plated in petri dishes and incubated in RPMI 1640 (Merck) supplemented with 10% fetal calf serum (Biowest, Nuaillé, France), 10 mM HEPES (Merck), 10 µg/ml penicillin/streptomycin (Merck), 1 mM sodium pyruvate (Merck), 2mM L-glutamine (Merck), and 15% M-CSF (supernatants of L929 mouse fibroblasts), for 7 days at 37 °C and 5% CO_2_. After 5 days, fresh medium was added. Cells were plated at a density of 8 × 10^4^ cells per well in 96-well plates. Mouse macrophages were either infected with *Mtb*, as indicated in the figure legends, or stimulated with LPS (5 µg/ml, 4 h at room temperature (RT); Sigma) and/or Nigericin (5 µM, 2 h at RT, Sigma).

### Cell culture

THP-1 macrophages (ATCC, Manassas, USA) were grown in RPMI medium supplemented with 10% FBS. MRC-5 human lung fibroblasts (Coriell Institute for Medical Research) were cultured in Minimum Essential Medium (MEM) supplemented with 10% FBS, 1% non-essential amino acids, and 1% sodium pyruvate. J774.2 macrophages (Merck) were grown in Dulbecco’s Modified Eagle Medium supplemented with 10% FBS. All cell lines were tested routinely for mycoplasma contamination.

### Culture conditions of bacteria

The mycobacterial strain Erdman (provided by S.T. Cole, Institute Pasteur Paris) was grown in Middlebrook 7H9 broth, supplemented with 10% albumin dextrose catalase, 0.05% Tween-80, and 0.2% glycerol.

### Survival assays

For the survival assays, macrophages were seeded in 96-well plates and preincubated with different compounds for 2 h. Primary human macrophages (multiplicity of infection (MOI) 1 or 2) and mouse BMDMs (MOI 3) were infected with *Mtb* Erdman in RPMI medium for 48 h. Afterwards, the cells were washed several times with phosphate-buffered saline (PBS) and fixed with 4% paraformaldehyde (PFA). Survival was assessed using 4’,6-diamidino-2-phenylindole (Thermo Fisher Scientific) staining. Images were acquired on an IX81 inverted microscope (Olympus, Hamburg, Germany) using the cellSens standard software (Olympus) and Fiji processing software to count the number of surviving cells, which was used to calculate cell survival.

### TMRM staining

Primary human macrophages, mouse BMDMs, and THP-1 wild-type and knock-out cells were seeded in a 96-well plate and infected with *Mtb* Erdman for up to 24 h at an MOI of 1, 2, and 3 respectively. For staining with TMRM, cells were washed several times with PBS before adding TMRM at a final concentration of 100 nM (Merck). Cells were incubated at 37 °C in 5% CO_2_ for 20 min and washed with PBS. Images were acquired on an IX81 inverted microscope using the cellSens software.

### SYTOX Green staining

Primary human macrophages were seeded in a 96-well plate and infected with *Mtb* Erdman for up to 24 h at an MOI of 1. For staining with SYTOX Green, cells were washed several times with PBS and stained with SYTOX Green at a final concentration of 100 nM (Thermo Fisher Scientific). Cells were incubated at 37 °C in 5% CO_2_ for 20 min and washed with PBS. Images were acquired on an IX81 inverted microscope using the cellSens software. Sytox-positive cells were counted to determine cell survival as indicated in the figure.

### Immunofluorescence staining

Primary human macrophages were seeded on 8-well chamber slides and infected with *Mtb* Erdman for 5 h at an MOI of 2. For GSDMD staining, cells were washed with PBS and stained with MitoTracker™ Red CMXRos (Thermo Fisher Scientific) according to the manufacturer’s recommendation. Cells were fixed with 4% PFA for 20 min at RT, washed three times with PBS, and permeabilized with 0.4% Triton X-100 and 0.1% Tween 20 in PBS. After three washes in PBS, cells were incubated with blocking solution (5% FBS, 0.1% Triton X-100, 0.1% Tween 20 in PBS) for 1 h at RT, followed by an overnight incubation at 4 °C with primary antibody (anti-GSDMD, HPA044487 from Merck; RRID: AB_2678957). After three washes in PBS, cells were incubated with the secondary antibody (4421 Cell Signaling Technology) for 1 h at RT. Finally, slides were mounted (ProLong™ Gold Antifade Mountant) and images were acquired using a Olympus Fluoview FV 1000 confocal microscope with ×60 objective. Magnification, microscope settings, and laser intensity were maintained equal for the different experimental conditions.

### Isolation of mitochondria

Mitochondria were isolated from 2 × 10^7^ primary human macrophages infected with *Mtb* Erdman (MOI 2) for 5 h using the Mitochondria Isolation Kit for Cultured Cells according to the manufacturer’s recommendations (Thermo Fisher Scientific). In detail, we used the reagent-based methods described in the manufacturer’s manual. The mitochondria pellet was lysed with 2% CHAPS (Thermo Fisher Scientific) in Tris-buffered saline (TBS; 25 mM Tris, 150 mM NaCl, pH 7.2), containing Halt™ Protease and Phosphatase Inhibitor Cocktail (Thermo Fisher Scientific) for 1 min by vortexing. Upon centrifugation, supernatants were harvested and the protein concentration was measured with the Pierce™ BCA Protein Assay Kit (Thermo Fisher Scientific) and mitochondrial lysates were subjected to immunoblot analysis.

### Immunoblot analysis

THP-1 monocytes were seeded in 6-well plates at a density of 4.5 × 10^6^ per well and incubated with 200 nm phorbol 12-myristate 13-acetate (PMA) for 72 h. Afterwards, the medium was changed to RPMI/FBS without PMA for 24 h. The cells were infected with *Mtb* Erdman at an MOI of 2 and supernatants were collected 24 h post infection. The supernatants were precipitated using methanol and chloroform and resuspended in Laemmli buffer. Proteins were subjected to sodium dodecyl sulfate-polyacrylamide gel electrophoresis and immunoblot analysis as previously described. The following antibodies were used: anti-ANT2 (14671; RRID: AB_2798562), anti-β-actin (4970), anti-cleaved IL-1β (83186; RRID: AB_2800010), and anti-NLRP3 (15101; RRID: AB_2722591) from Cell Signaling Technology and anti-GSDMD (HPA044487; RRID: AB_2678957) from Merck.

### Enzyme-linked immunosorbent assay (ELISA)

IL-1β and IL-18 ELISA (BioLegend, San Diego, USA) were performed according to the manufacturer’s recommendations. BMDMs, primary macrophages, and THP-1 cells were used for this assay and infected as described before. For ESX-1 inhibition assay 2.5 × 10^6^ THP-1 GSDMD knock-out cells were seeded per well infected with an MOI of 1. Five hours post infection, BTP-15 (10 µM) was added to the cells for additional 24 h. Lysates and supernatants were taken as described before 5 h and in total 29 h post infection. Briefly, supernatant obtained from human macrophage and THP-1 cells was diluted 1:10 and supernatant from BMDMs was diluted 1:2 in the ELISA Kit diluent and incubated for 2 h. All samples were measured in technical duplicates and the concentration was determined with the corresponding standard curve. The optical density was measured in a Hidex Sense microplate reader (Hidex, Turku, Finland), and the data were analyzed with the GraphPad Prism 8.0.2 software (GraphPad, San Diego, CA, USA).

### Proteomics

Whole-cell lysates were gained from uninfected and *Mtb*-infected J774.2 macrophages (MOI 5) 24 h post infection. The protein concentration was determined by the Pierce™ BCA Protein Assay Kit and 50 µg protein was precipitated using ice-cold acetone. Chromatin degradation was achieved in a Bioruptor (10 min, cycle 30/30 s) in 8 M urea buffer in 50 mM triethylammoniumbicarbonate (TEAB). Digestion was carried out via an in-solution digest. Briefly, proteins were reduced with 5 mM dithiothreitol for 1 h at 25 °C and alkylated with 40 mM chloroacetamide for 30 min at RT in the dark. Lys-C was added at a 1-to-75 enzyme-to-substrate ratio for pre-digestion for 4 h at 25 °C. Afterwards, 50 mM TEAB was used to achieve a urea concentration of 2 M for digestion with 1 µg/µl trypsin. Samples were incubated overnight, and digestion was stopped by acidification with 1% formic acid. Finally, the samples were primed by STAGE tip technique and analyzed by mass spectrometry using data-independent acquisition. Mass spectrometry and data analysis were conducted by Dr. Stefan Müller at the Proteomics Core Facility Cologne.

### Quantification of ROS

MRC-5 fibroblasts were seeded in black 96-well plates at a density of 2 × 10^4^ cells per well and infected with *Mtb* Erdman at an MOI of 10. Mitochondrial ROS production was analyzed by MitoSOX Red (Thermo Fisher Scientific) 24 h post infection. The cells were washed with Hank’s Balanced Salt Solution (HBSS) and incubated with MitoSOX Red (5 μM in HBSS) at 37 °C for 15 min. Following incubation, cells were washed three times with HBSS and fluorescence was detected in a BioTek Cytation™ 3 Cell Imaging Multi-Mode Reader (Bad Friedrichshall, Germany). The cell-specific fluorescence was calculated by subtracting the fluorescence of cell-free wells containing HBSS.

### Quantification of calcium

Intracellular calcium concentration was determined using the Fluo-4 NW Calcium Assay Kit according to the manufacturer’s instructions. Briefly, J774.2 macrophages were plated at a density of 2 × 10^4^ cells per well in black-walled 96-well plates and infected with *Mtb* Erdman at an MOI of 5 for up to 24 h. Cells were washed with HBSS and incubated with Fluo-4 NW for 30 min at 37 °C. Fluo-4 NW (excitation wavelength/emission wavelength: 488 nm/530 nm) was detected using a BioTek Cytation™ 3 Cell Imaging Multi-Mode Reader. Calcium was quantified by calculating the ratio of the ion-bound (excitation wavelengths 340 nm) and ion-free indicators (excitation wavelengths 380 nm).

### *Mtb* infection of mice

IL-13-overexpressing (tg) mice on a BALB/c genetic background [34] and BALB/c heterozygous littermates were bred under specific-pathogen-free conditions in the animal facilities of the Research Center Borstel. For infection experiments, age- and sex-matched animals were transferred to the BSL3 facility and kept in individually ventilated cages. Mice were infected with an aerosol containing the *Mtb* strain H37Rv using an inhalation exposure system (Glas-Col, Terre-Haute, IN, USA) as described [34]. All animal experiments were performed in accordance with the German Animal Welfare Act and approved by the Ministry of Energy, Agriculture, the Environment, Nature and Digitalization, Kiel, Germany (approval number 33-05/04).

### Immunohistochemistry of the lungs

Lungs were fixed in 4% buffered formalin, embedded in paraffin, and sectioned on a microtome. To examine histopathological changes during infection, sections were stained with hematoxylin/eosin.

### Quantitative real-time RT-PCR

Before and at various time points of infection, pieces of lungs were weighed and homogenized in 4 M guanidinium isothiocyanate buffer. After acid phenol extraction, RNA was transcribed into cDNA and qPCR was performed using a light cycler (Roche Diagnostics Corporation, Indianapolis, IN USA). The following primer and probe sets were employed. *Hprt*: sense 5′-TCC TCC TCA GAC CGC TTT T-3′, antisense 5′-CCT GGT TCA TCA TCG CTA ATC-3′, probe 5′-AGT CCA G-3′; *Nlrp3*: sense 5′- -3′, antisense 5′- -3′, probe 5′- -3′.

### Generation of THP-1 knock-out cells

All THP-1 lines were cultured as previously described. In all, 1 × 10^6^ THP-1 cells were spin infected (800 rpm, 2 h, 37 °C) twice with third-generation Lentiviral Cas9-EGFP (ThermoFisher) particles (MOI 5) and eGFP^high^-expressing single-cell clones were selected by eGFP-mediated fluorescence-activated cell sorting. Lentiviral particles containing guide RNA were purchased from ThermoFisher and used according to the manufacturer’s procedure (four separate guide RNA constructs were used for each knock-out). In all, 10^6^ THP-1 Cas9-GFP cells were transduced with lentiviral particles (each guide separate and in combination, MOI 5). Spin-infection was performed at 800 rpm for 2 h. Centrifuge was heated during that time to 37 °C and media was supplemented with 20 mM Hepes (ThermoFisher). Twenty-four hours post infection, media was replenished and cells were cultured in media as described. Selection was performed by addition of puromycin (Roth) into the culture media. Specific gene knock-out was validated via immunoblotting.

### Statistical analysis

Data are expressed as mean ± standard error of the mean (SEM). The data were analyzed with the Graphpad Prism version 8.0.2 software program. Mann–Whitney *U*-Test was used to compare two matched groups and the one-way analysis of variance with Bonferroni post-tests was used to compare more than two groups and differences with *p* values of <0.05 were considered to be statistically significant. No data were excluded from the analysis of experiments.

## Supplementary information

Supplement

## Data Availability

The authors declare that all data supporting the findings of this study are available within the paper and its Supplementary Information files. Proteom data was uploaded the the public database PRIDE. Project accession number: PXD029017.
