# Supplementary material for: Gasdermin D mediates host cell death but not interleukin-1β secretion in Mycobacterium tuberculosis-infected macrophages
Source: Cell Death Discov. 2021 Oct 30;7:327. doi: 10.1038/s41420-021-00716-5 (PMC8557205; doi:10.1038/s41420-021-00716-5)
Supplement: Supplementary file 1 — Supplement [file 41420_2021_716_MOESM1_ESM.docx]

**Supplementary information for:**

**Gasdermin D mediates host cell death but not interleukin-1β secretion in *Mycobacterium tuberculosis* infected macrophages**

**Supplement Fig. 1 a** Proteome analysis of whole cell lysates by mass spectrometry of uninfected and *Mtb*-infected J774.2 macrophages (Mᴓ; n=3). **b** Intracellular calcium (Ca^2+^) concentration in *Mtb*-infected J774.2 Mᴓ (MOI 5) in the presence or absence of Ru-360 (n=16). **c** Survival of *Mtb*-infected primary human Mᴓ treated with Ru-360 48 h post infection using DAPI staining (n=8). Data from two experiments with multiple replicates are shown in **b** and **c**. Results are expressed as mean ± SEM. Analysis was done using One-Way ANOVA with Bonferroni post-test (***, p≤ 0.001; ****, p≤ 0.0001). **d** Representative microscope pictures (DAPI, blue) from **c** using primary human macrophages infected with *Mtb* (MOI 2, 48h post infection) and pre-treated with DMSO, RIF, Mitotempo, Ru-360 and Mitotempo/Ru-360 (scale bar: 100 μm). **e** Representative microscope pictures (DAPI, blue) from BCL-2 overexpressing BMDM (w/o and with CRE treatment) infected with *Mtb* (MOI 3, 24h post infection. Cells were pre-treated with DMSO and rifampicin (RIF) as indicated (scale bar: 100 μm). **f** Quantification of cleaved IL-1β (24h post infection) in the supernatant of *Mtb* infected macrophages and treated with MitoTEMP (n=2).

**Supplement Fig. 2 a** Representative microscope pictures (DAPI, blue) from *Mtb* (MOI 1, 24h post infection) infected primary human macrophages treated as indicated with DMSO, rifampicin (RIF), MCC950 and VX-765 (scale bar: 100 μm). **b** Representative microscope pictures (DAPI, blue) from *Mtb* (MOI 1, 24h post infection) infected primary human macrophages treated as indicated with DMSO, rifampicin (RIF), MCC950 and VX-765 (scale bar: 100 μm). **c** Quantification of cleaved IL-1β (5h and 24h post infection) in the supernatant of *Mtb* infected macrophages and treated with MCC-950 (n=2). **d** Quantification of NLRP3 (5h and 24h post infection) in the total cell lysates of *Mtb* infected macrophages (n=2). Relative protein expression was calculated in relation to the β-actin loading control.

**Supplement Fig. 3 a** IL-1β concentration (pg/ml) in the supernatant of wild-type (black circle) and GsdmdD276A BMBM (white circle) stimulated with LPS (5 µg/ml) and/or Nigericin (5 µM) as indicated in the figure (n=4 for both groups). Results are expressed as mean ± SEM. Analysis was done using One-Way ANOVA with Bonferroni post-test (**, p≤ 0.01). **b** Western blot analysis of whole cell lysates showing GSDMD and NLPR3 knock-out (*ko*) THP-1 cells compared to wild-type (*wt*), scrambled (*scr*) and HBRT (*HBRT*). β-actin was used as loading control as indicated. **c** Representative microscope pictures (TMRM staining, red) from *Mtb* (MOI as indicated, 24h post infection) infected wild-type, GSDMD and NLPR3 knock-out THP-1 macrophages (scale bar: 100 μm). **d** IL-1β (pg/ml) of LPS (5 µg/ml) and/or Nigericin (5 µM) stimulated wt, NLPR3 and GSDMD knock-out THP-1 cells (n=3). Results are expressed as mean ± SEM. Analysis was done using One-Way ANOVA with Bonferroni post-test (****, p≤ 0.0001).

**
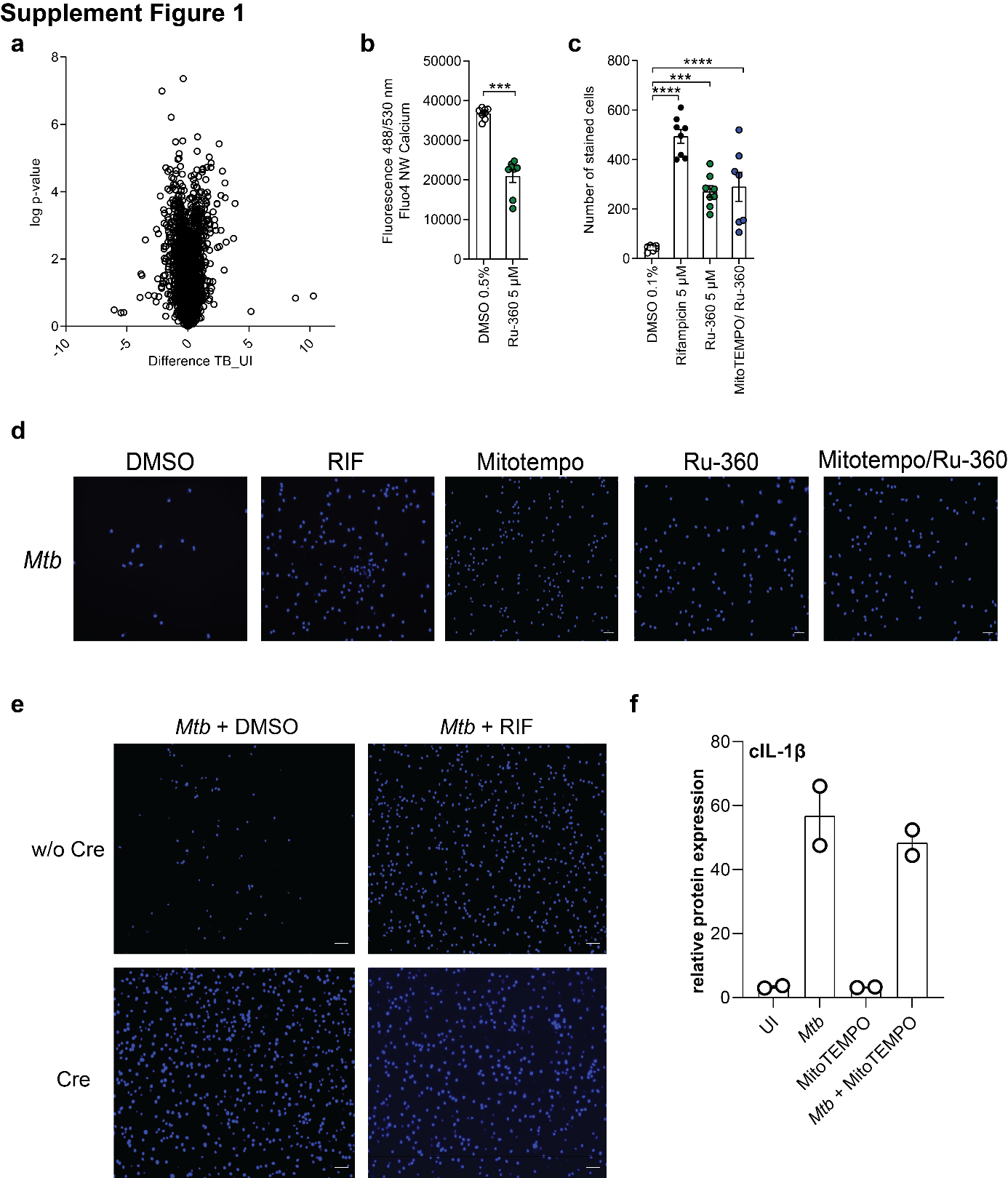
**

**
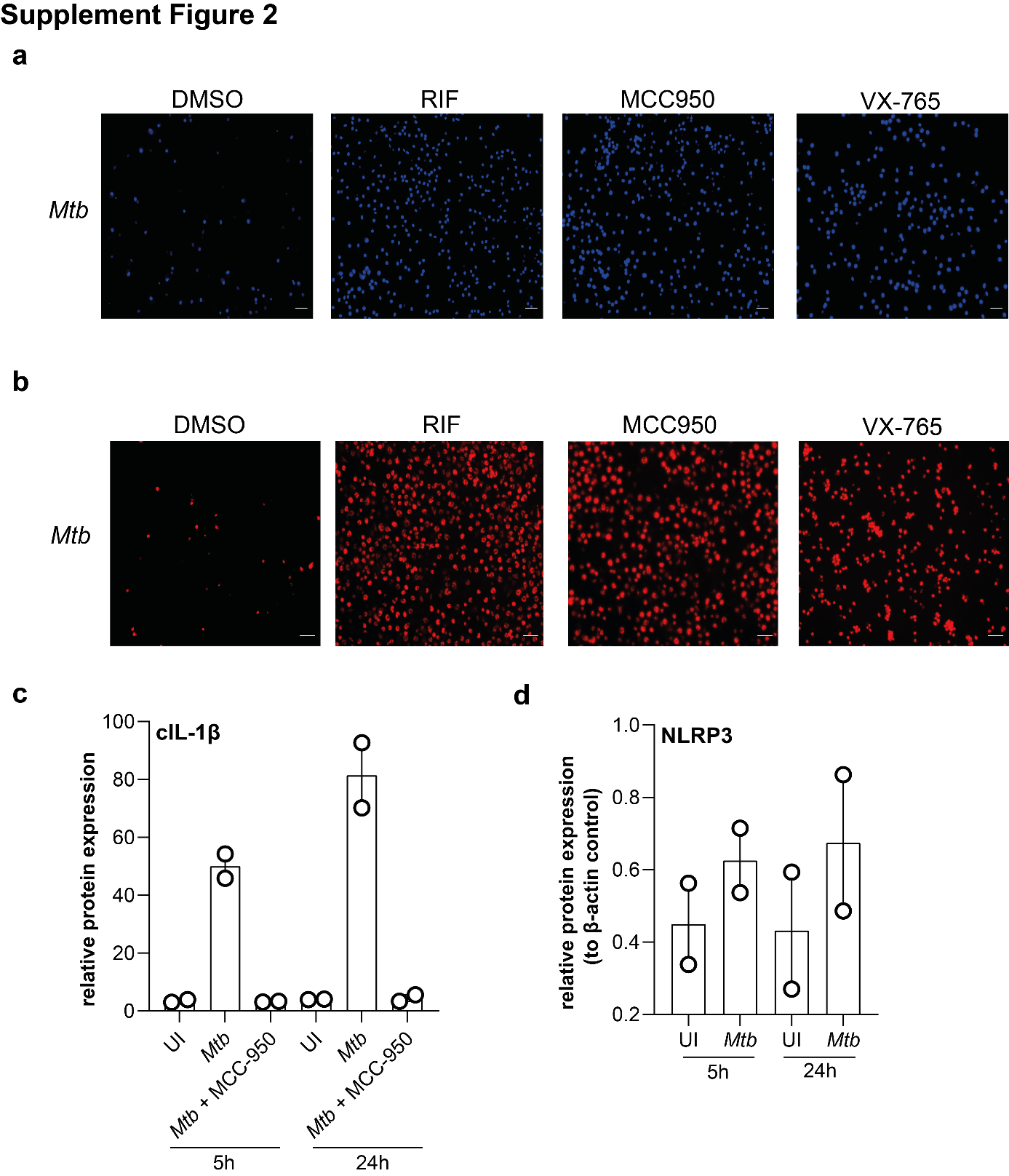
**

**
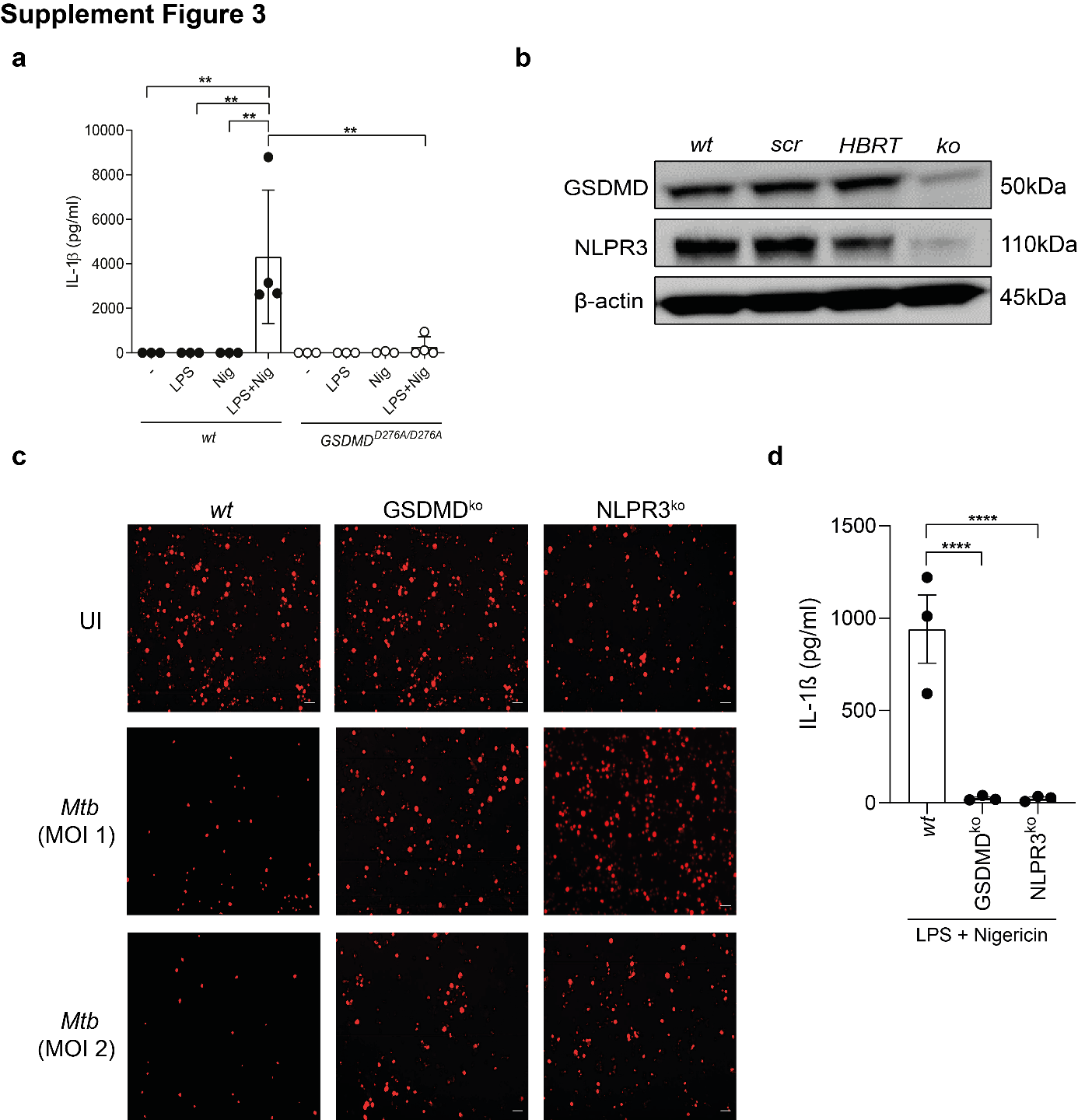
**
